# A Novel Plant-Derived Choline Transporter-like Protein 1 Inhibitor, Amb544925, Induces Apoptotic Cell Death via the Ceramide/Survivin Pathway in Tongue Squamous Cell Carcinoma

**DOI:** 10.3390/cancers14020329

**Published:** 2022-01-10

**Authors:** Kaoru Shibata, Nozomi Nishijima, Kaho Hirai, Saiichiro Watanabe, Tsuyoshi Yamanaka, Daichi Chikazu, Masato Inazu

**Affiliations:** 1 Department of Oral and Maxillofacial Surgery, Tokyo Medical University, 6-7-1 Nishishinjuku, Shinjuku-ku, Tokyo 160-0023, Japan; kshibata@tokyo-med.ac.jp (K.S.); chikazu@tokyo-med.ac.jp (D.C.); 2 Institute of Medical Science, Tokyo Medical University, 6-1-1 Shinjuku, Shinjuku-ku, Tokyo 160-8402, Japan; s22061193@stu.rakuno.ac.jp (N.N.); s118043@tokyo-med.ac.jp (K.H.); m270119s@tokyo-med.ac.jp (S.W.); 3 Department of Molecular Preventive Medicine, Tokyo Medical University, 6-1-1 Shinjuku, Shinjuku-ku, Tokyo 160-8402, Japan; yamanaka0244@gmail.com

**Keywords:** choline transporter, tongue squamous cell carcinoma, apoptosis, ceramide, survivin, metastasis

## Abstract

**Simple Summary:**

In many cancer cells, the choline uptake mechanism via the choline transporter-like protein 1 (CTL1) is involved in cell proliferation, and inhibiting its function is known to induce cell death by apoptosis. Therefore, CTL1 is attracting attention as a target molecule for cancer therapy. Here, we investigated the mechanism of the antitumor effect and metastasis inhibition of Amb544925, a novel CTL1 inhibitor discovered from plant-derived organic compounds in the tongue squamous cell carcinoma (TSCC) cell line, HSC-3. Amb544925 increased caspase-3/7 activity and caused apoptotic cell death. The antitumor effect of Amb544925 was brought about by suppressing the expression of survivin, an apoptosis inhibitor, through ceramide, an apoptosis-inducing factor (ceramide/survivin pathway). Furthermore, it was suggested that the inhibitory effect of Amb544925 on cell migration was also mediated by the ceramide/survivin pathway. Plant-derived Amb544925 is a lead compound in the treatment of TSCC that exerts antitumor effects via the ceramide/survivin pathway by targeting CTL1.

**Abstract:**

Background: Despite recent advances in the early detection and treatment of TSCC patients, recurrence rates and survival rates have not improved. The high frequency of lymph node metastasis is one of the causes, and the drug development of new therapeutic mechanisms such as metastasis control is desired. Choline transporter-like protein 1 (CTL1) has attracted attention as a target molecule in cancer therapy. In this study, we examined the antitumor effects of Amb544925, a plant-derived CTL1 inhibitor. Methods: The TSCC cell line HSC-3 was used to measure [^3^H]choline uptake, cell survival, caspase activity, and cell migration. Xenograft model mice were prepared to verify the antitumor effect of Amb544925. Results: Amb544925 inhibited cell viability and increased caspase-3/7 activity at concentrations that inhibited choline uptake. Amb544925 and ceramide increased SMPD4 expression and suppressed surivivin expression. Furthermore, Amb544925 and ceramide inhibited the migration of HSC-3 cells. In the xenograft model mice, Amb544925 suppressed tumor growth and CTL1 mRNA expression. Conclusions: The plant-derived CTL1 inhibitor Amb544925 is a lead compound of a new anticancer agent exhibiting antitumor effects and inhibition of cell migration through the ceramide/survivin pathway.

## 1. Introduction

Oral squamous cell carcinoma (OSCC) is the most frequently occurring malignancy of the oral cavity [1]. Despite advances in treatment, there has been no significant improvement in prognosis. Causes for this include its high metastatic potential and resistance to radiation therapy and chemotherapy [1]. Tongue squamous cell carcinoma (TSCC) is the most common type of OSCC and is a highly aggressive malignant cancer with lymph node and distant organ metastasis, resulting in a low survival rate. TSCC is thought to be triggered by chronic chemical irritants such as alcohol consumption and smoking, as well as chronic mechanical irritants such as the constant contact of misaligned teeth. The number of TSCC cases has been increasing in recent decades [2], and TSCC is more common in young adult female patients [3,4]. A high frequency of cervical lymph node metastasis, a high risk of recurrence, and resistance to chemotherapy are the characteristics of TSCC [5]. Despite recent advances in the early detection and treatment of patients with TSCC, recurrence rates and survival rates have not improved over the past decade, with 5-year survival rates of only 50%–60%. TSCC first metastasizes to regional lymph nodes and then reaches the lungs. The presence of lymph node metastasis reduces the survival rate by about half [1,6]. One of the reasons for the lack of improvement in the survival rate of TSCC is the lack of therapeutic interventions for lymph node metastasis. In order to meet unmet medical needs, there are expectations for the development of new therapeutic agents with metastasis inhibitors and novel therapeutic mechanisms.

Choline is water-soluble and exists in the body as a quaternary ammonium ion, and is one of the biofactors ingested from the diet. The main physiological function of choline is that it is used as a precursor for the neurotransmitter acetylcholine, and functions as a donor of methyl groups. Furthermore, choline is used as a precursor for phosphatidylcholine (PCho) and sphingomyelin, which are phospholipids in cell membranes, and is also essential during cell division, when cell membrane synthesis is active. Cancer cells actively take up choline and use it for cell proliferation. Taking advantage of these properties, positron emission tomography (PET) imaging with ^14^C-choline and ^18^F-choline has been performed in various cancer patients [7,8,9]. Therefore, understanding the mechanism of choline uptake in cancer cells may lead to the proposal of new therapeutic mechanisms.

The choline transporters are responsible for choline uptake into the cell and are currently classified into three groups [10]. The first is the high-affinity choline transporter 1 (CHT1), which is expressed in cholinergic neurons and is linked to the synthesis of ACh. The second is the choline transporter-like protein (CTL) family, which has five subtypes (CTL1-5). These transporter proteins differ in their tissue distribution and choline transport properties and are deeply involved in the physiological functions of each tissue. Third, only the organic cation transporters (OCTs), OCT1 and OCT2, recognize and transport choline. However, they have a very low affinity for choline. We previously elucidated the mechanism of choline transport in various cancer cell lines and have identified transporters that play a role in choline transport [11,12]. We also found that extracellular choline uptake in TSCC cell line HSC-3 is mediated by CTL1, and its functional inhibition induces cell death by means of apoptosis [13]. This CTL1-mediated choline transport system is a potential new target for the treatment of TSCC.

Plants produce a wide variety of secondary metabolites, which are estimated to be present in one million species [14]. Most of them are unknown substances and are highly anticipated as sources for drug discovery. Currently, plant-derived secondary metabolites are being clinically applied as anticancer agents. Plant alkaloids with anticancer properties have been discovered and extracted, and microtubule inhibitors such as vincristine and docetaxel, as well as topoisomerase inhibitors such as irinotecan and etoposide, are used in many cancer treatments. Therefore, we discovered Amb4269951 and its derivative Amb4269675, which inhibit CTL1 function and suppress cancer cell survival, from a library of organic compounds derived from plants [11,12]. These compounds inhibited CTL1-mediated choline uptake in glioma cells and pancreatic cancer cell lines, suggesting that they induce cell death by apoptosis. Unfortunately, both compounds are cationic compounds and have little potential as pharmaceuticals due to difficulties in stability and chemical synthesis. In our search for plant-derived organic compounds with CTL1 inhibitory activity, we discovered Amb544925, a novel non-cationic compound. 

In this study, we elucidated the antitumor effect of Amb544925, a novel non-cationic compound, on HC-3 cells and its mechanism. In addition, the inhibitory effect on metastasis was also verified.

## 2. Materials and Methods

### 2.1. Cells and Cell Culture

TSCC cell line HSC-3 was provided by the Japanese Collection of Research Bioresources Cell Bank (Cell Registration No. JCRB0623, Osaka, Japan). The HSC-3 cell line was established from a tumor in a metastatic lymph node derived from a squamous cell carcinoma of the tongue in a 64-year-old male patient and is a suitable model for the study of metastatic squamous cell carcinoma. HSC-3 cells were grown in RPMI 1640 medium (FUJIFILM Wako Pure Chemical Corporation, Osaka, Japan) supplemented with 10% fetal bovine serum (FBS) (Biowest SAS, Nuaille, France) and penicillin G (100 units/mL) and streptomycin (100 µg/mL) on CELLSTAR^®^ Standard Cell Culture Flasks (Greiner Bio-One GmbH, Frickenhausen, Germany). Cultures were maintained in a humidified atmosphere of 5% CO_2_ and 95% air at 37 °C, and the medium was changed every 3–4 days.

### 2.2. Isoquinoline Derivative Amb544925

Amb544925 (1-(4-methoxy-6-methyl-5,6,7,8-tetrahydro-[1,3]dioxolo[4,5-g]isoquinolin-9-yl)-4-methyl-5-phenyl-penta-2,4-dien-1-one) was provided from Greenpharma SAS (Orleans, France) and is an organic compound extracted from the opium poppy (*Papaver somniferum* L.). Amb544925 was dissolved in DMSO as a solvent and used in the experiment at a final concentration of 1%. Greenpharma Natural Compound Library was purchased from Greenpharma SAS (Orleans, France), and contains compounds representative of many phytochemical families.

### 2.3. [^3^H]choline Uptake in HSC-3 Cells

The [^3^H]choline uptake experiments were measured using previously established methods [13]. [Methyl-^3^H]choline chloride (NET109, 2864 GBq/mmol) was purchased from PerkinElmer, Inc. (Waltham, MA, USA). HSC-3 cells were cultured in CELLSTAR^®^ 24 Well Cell Culture Multiwell Plates (Greiner Bio-One GmbH, Frickenhausen, Germany). The medium was removed, and the cells were washed twice with uptake buffer (125 mM NaCl, 4.8 mM KCl, 1.2 mM CaCl_2_, 1.2 mM MgSO_4_, 1.2 mM KH_2_PO_4_, 5.6 mM glucose and 25 mM HEPES, pH 7.4). After pretreatment with each concentration of Amb544925, 10 µM [^3^H]choline uptake for 20 min was performed. The cells were washed twice with ice-cold uptake buffer, and then lysed with 0.1% Triton X-100. The cell lysate was mixed with Hionic-Fluor (PerkinEimer, Inc., Waltham, MA, USA), and the radioactivity was measured using a liquid scintillation counter (Tri-Carb1 2100 TR, Packard Instrument Company, Meriden, CT, USA).

### 2.4. Measurement of Viable Cells

Cell viability was measured according to previously established methods [11,12,13,15,16]. Cells were plated in 24-well plates and cultured under various conditions, including treatment with inhibitors (Amb544925, C2 ceramide and hemicholinium-3 (HC-3)). C2 ceramide was purchased from Cayman Chemical (Ann Arbor, MI, USA). HC-3 was purchased from Sigma-Aldrich Co. LLC (St. Louis, MO, USA). Cell numbers were determined using the CellTiter-Glo^®^ Luminescent Cell Viability Assay (Promega, Madison, WI, USA). Luminescence intensity was measured with a FilterMax F5 Multi-Mode Microplate Reader (Molecular Devices, LLC, Sunnyvale, CA, USA).

### 2.5. Measurement of Caspase 3/7 Activity

Caspase 3/7 activity was measured according to previously established methods [11,12,13,15,16]. Cells were seeded in 24-well plates and cultured under various conditions, including treatment with inhibitors (Amb544925, C2 ceramide and HC-3). Caspase-3/7 activity and cell number were simultaneously measured using the Caspase-Glo^®^ 3/7 Assay (Promega, Madison, WI, USA) and the CellTiter-Glo^®^ Luminescent Cell Viability Assay (Promega, Madison, WI, USA). Luminescence was measured with a FilterMax F5 Multi-Mode Microplate Reader (Molecular Devices, LLC, Sunnyvale, CA, USA). Caspase 3/7 activity was calculated as the activity per number of cells.

### 2.6. Quantification of mRNA Expression Levels by Reverse Transcription-Polymerase Chain Reaction (RT-PCR)

Quantification of mRNA expression levels was measured according to a previously established method [11,12,13]. Extraction of total RNA from cells was performed using QIAshredder and RNeasy Mini Kit (QIAGEN, Valencia, CA, USA). Real-time PCR was performed using a TaqMan^®^ Gene Expression Assays (Applied Biosystems, Foster City, CA, USA) and TaqMan^®^ RNA-to-CT^TM^ 1-Step Kit (Applied Biosystems, Foster City, CA, USA). The assay IDs of the TaqMan probes used were: CTL1, Hs00223114_m1; CTL2, Hs01105936_m1; SMPD1, Hs03679345_m1; SMPD2, Hs00906924_g1; SMPD3, Hs00920354_m1; SMPD4, Hs004187047_g1; SMPDL3A, Hs01041066_m1; SMPDL3B, Hs01038741_m1; survivin, Hs00977612_mL; glyceraldehyde-3-phosphate dehydrogenase (GAPDH), Hs99999905_mL; human β-actin, Hs99999903_mL; mouse CTL1, Mm01350815_m1; and mouse β-actin, Mm00607939_s1. RT-PCR was performed using the LightCycler^®^ 96 system (Roche Diagnostics, Mannheim, Germany). Relative expression was calculated as the expression level of the target gene relative to the housekeeping gene using the ΔCt method [13,15,16].

### 2.7. Western Blotting Analysis

Western blotting analysis was measured according to a previously established method [11,12,13]. Cells were lysed by sonication on ice using radioimmunoprecipitation lysis buffer (Santa Cruz Biotechnology, Inc., Dallas, TX, USA) containing 1 mM ethylenediaminetetraacetic acid and a cocktail of protease inhibitors (Thermo Fisher Scientific Pierce Biotechnology, Rockford, IL, USA). The cell lysate was placed on ice for 10 min and then centrifuged (14,000× *g*) at 4 °C for 15 min. Samples were electrophoresed in Any kD^TM^ Mini-PROTEAN^®^ TGX^TM^ Gel (BioRad Laboratories, Inc., Hercules, CA, USA) with molecular weight standards (DynaMarker Protein MultiColor III, BioDynamics Laboratory Inc., Tokyo, Japan) using the Trans-Blot^®^ Turbo^TM^ Transfer System (BioRad Laboratories, Inc., Hercules, CA, USA). PVDF membranes were blocked with iBind^TM^ Flex Solution (Thermo Fisher Scientific Inc., Waltham, MA, USA) overnight at 4 °C, and then incubated with the following primary antibodies overnight at 4 °C. For the detection of SMPD4, survivin and GAPDH, anti-SMPD4 antibody (4 μg/mL, Cat. No. ab133935, Abcam plc, Cambridge, UK), anti-servivin antibody (4 μg/mL, Cat. No. ab76424, Abcam plc, Cambridge, UK) and anti-GAPDH mouse antibody (1:1000, Cat. No. M171-3, Medical & Biological Laboratories Co., LTD., Nagoya, Japan) were used, respectively. PVDF membranes were washed three times with iBind^TM^ Flex Solution and then incubated with HRP-conjugated anti-rabbit IgG (1 μg/mL, Cat. No. 074-1506, Kirkegaard and Perry Laboratories Inc., Gaithersburg, MD, USA), HRP-conjugated anti-mouse IgG (1 μg/mL, Cat. No. 074-1806, Kirkegaard and Perry Laboratories Inc., Gaithersburg, MD, USA) or anti-β-actin pAb-HRP-DirecT (1:1000, Cat. No. PM053-7, Medical & Biological Laboratories Co., Ltd., Nagoya, Japan) as a secondary antibody for 1 h at room temperature. The bands were detected by the chemiluminescence signal using the ECL™ Prime Western Blotting Detection System. Luminescence images were acquired and quantified using the ChemiDoc™ XRS+ System with Image Lab™ 6.1 Software (Bio-Rad Laboratories, Hercules, CA, USA).

### 2.8. Migration Assay

Migration assay was measured by partially modifying a previously established method [17]. A suspension of HSC-3 cells was prepared to 5 × 10^5^ cells/mL and 70 μL per well was added to the ibidi^®^ culture insert (Culture–Insert 2 Well in μ–Dish 35 mm, low, ibidi, Martinsried, Germany). The cells were then incubated at 37 °C for 24 h until they became 100% confluent. The culture medium was removed and 5 μg/mL of mitomycin C (FUJIFILM Wako Pure Chemical Corporation, Osaka, Japan) was added to the insert to inhibit cell proliferation and incubated for 2 h. The culture insert was removed with sterile tweezers, and 1 mL of each concentration of Amb544925 was added to the culture dish, followed by incubation for 24 h. The migrated images were captured using the EVOS XL Core Imaging System (Thermo Fisher Scientific Inc., Waltham, MA, USA) and the cell-covered area was quantified using ImageJ software (version 1.53, National Institutes of Health, Bethesda, MD, USA).

### 2.9. Mouse Xenograft Model

The previously described xenograft tumor model in nude mice [11,12] was used with some modifications. All animal experiments at Tokyo Medical University were approved by the University Animal Experiment Ethics Committee (approval number H31-0044). To generate solid tumors of HSC-3 cells, a suspension of HSC-3 cells (1 × 10^7^ cell/mice) mixed with Corning^®^ Matrigel^®^ (Corning Incorporated, Corning, NY, USA) was injected subcutaneously into the dorsal side of 6-week-old male BALB/cAJcl-nu/nu mice (Clare Japan Co., Ltd., Shizuoka, Japan). The developed solid tumors were then excised, and 3 mm square blocks were made and implanted into the dorsal side of 6-week-old male nude mice. Drug administration was started when the tumor volume reached approximately 200 mm^3^. Tumor volume was calculated by measuring the long (L) and short (W) diameters of the tumor and multiplying by L × W^2^ × 1/2. The administration solution of Amb544925 was prepared with DMSO each time and stored at room temperature under light shielding until administration. Amb544925 was administered intraperitoneally (100 μL/mouse) once daily in the morning at a dose of 10 mg/kg for 8 consecutive days, and the solvent control group received 100% DMSO (100 μL/mouse). Before administering the drug, the weight of the mice was measured, and their condition was observed. On day 19, mice were dissected, and tumors, brain, liver, lungs, heart, stomach, small intestine, spleen, kidneys, and testicles were removed and stored in RNA later^®^ (Thermo Fisher Scientific Inc., Waltham, USA). Total RNA from the tumor and each tissue was extracted using ISOGEN^®^ (Nippon Gene Co., Ltd., Tokyo, Japan). CTL1 mRNA expression in each tissue was quantified by RT-PCR method as described above.

### 2.10. Data Analysis

Data were expressed as the mean value ± standard deviation. Statistical analysis was performed using the statistical analysis software Prism 8 (GraphPad Software, Inc., San Diego, CA, USA). Comparisons between two groups were performed by means of unpaired *t*-test, and comparisons between three or more groups were performed by means of one-way ANOVA with Dunnett’s multiple-comparisons test and two-way ANOVA with Sidak’s multiple-comparisons test. A *p*-value of less than 0.05 in any statistical analysis was considered statistically significant.

## 3. Results

### 3.1. Chemical Characteristics of Amb544925

**Figure 1:**
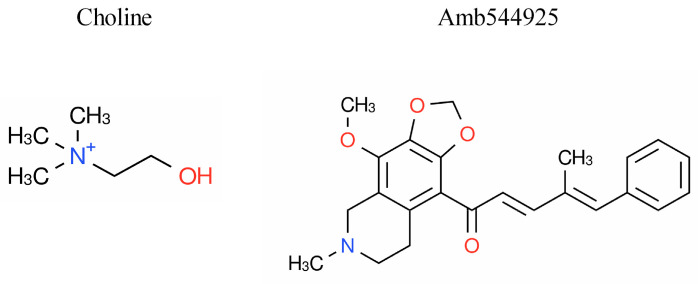
Chemical structure of choline (2-hydroxyethyl(trimethyl)ammonium), Amb544925 (1-(4-methoxy-6-methyl-5,6,7,8-tetrahydro-[1,3]dioxolo[4,5-g]isoquinolin-9-yl)-4-methyl-5-phenyl-penta-2,4-dien-1-one).

Amb544925 is an alkaloid extracted from the poppy (*Papaver somniferum* L.) and is an isoquinoline derivative with a monomethylamine group, whereas choline has a trimethylamine group. Both have a common moiety with a methylamine group (Figure 1). The chemical characteristics of Amb544925 include a molecular weight of 391.46, an octanol-water coefficient of distribution (LogP) value of 4.1921, and it is a lipophilic compound.

### 3.2. Effects of Amb544925 on Choline Uptake, Cell Survival and Caspase-3/7 Activity

**Figure 2:**
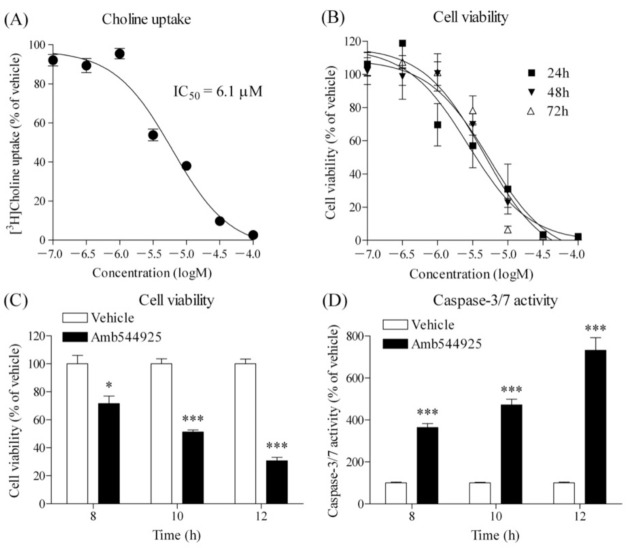
Effects of Amb544925 on [^3^H]choline uptake, cell viability and caspase 3/7 activity in HSC-3 cells. (**A**) Effect of Amb544925 on 10 µM [^3^H]choline uptake (*n* = 3). Cells were preincubated with each concentration of Amb544925 for 20 min, and then 10 µM [^3^H]choline uptake was added and uptake was measured for 20 min. The uptake of [^3^H]choline in the Amb544925 treatment was expressed as a percentage of that in the vehicle control. The IC_50_ value calculated from the four-parameter logistic curve was 6.1 μM. (**B**) Effect of Amb544925 on cell viability. Cells were incubated with each concentration of Amb544925 for 24, 48, and 72 h, and then cell viability was measured (*n* = 3). Results are presented as a percentage of the vehicle control. Effect of 30 µM Amb544925 on cell viability (**C**) and caspase-3/7 activity (**D**). Cells were incubated with 30 µM Amb544925 for 8, 10, and 12 h, and then cell viability and caspase-3/7 activity were measured simultaneously (*n* = 4). The results were presented as a percentage of the vehicle control. * *p* < 0.05 and *** *p* < 0.005 compared to the vehicle control.

First, we examined the effect of Amb544925 on [^3^H]choline uptake in HSC-3 cells (Figure 2A). Amb544925 inhibited choline uptake in a concentration-dependent manner with an IC_50_ value of 6.1 µM. We then examined the effect of Amb544925 on cell viability in HSC-3 cells (Figure 2B). Amb544925 inhibited cell survival in a concentration-dependent manner, similar to the concentration range of choline uptake inhibition. The growth inhibition effect was not enhanced by increasing the treatment time and was at its maximum at 24 h. The effects of Amb544925 on cell viability (Figure 2C) and caspase-3/7 activity (Figure 2D) at treatment times of 8, 10, and 12 h were examined simultaneously. Amb544925 significantly inhibited the cell viability of HSC-3 cells and significantly increased caspase-3/7 activity, and both effects were inversely correlated. Amb544925 inhibited the cell viability of various cancer cell lines in a concentration-dependent manner (Figure S1).

### 3.3. Effect of Amb544925 on the Expression of Sphingomyelinases

**Figure 3:**
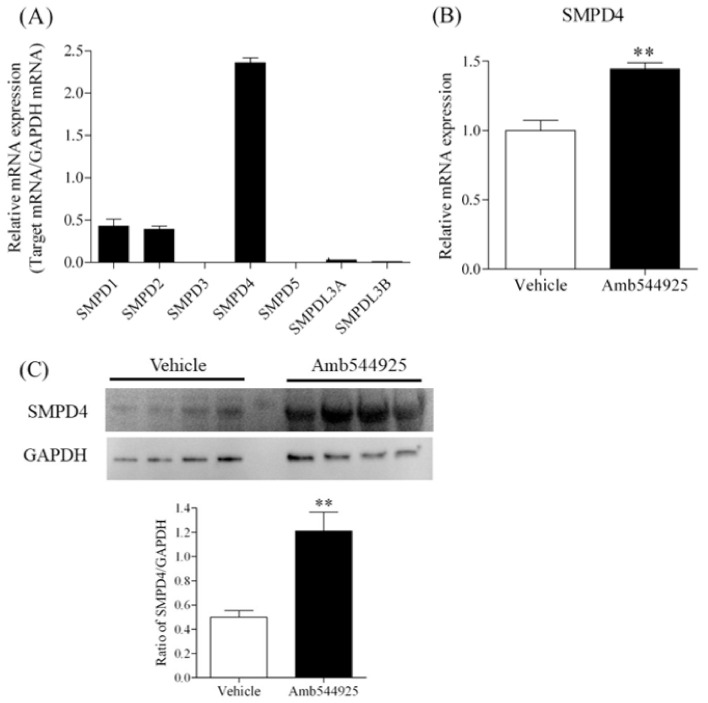
Effect of Amb544925 on SMPD4 expression in HSC-3 cells. (**A**) The mRNA expression patterns of seven different SMPD isoforms in HSC-3 cells (*n* = 3). Relative mRNA expression is expressed as the ratio of each target mRNA to the amount of GAPDH mRNA. (**B**) Effect of Amb544925 on SMPD4 mRNA expression in HSC-3 cells (*n* = 3). Cells were preincubated with 30 µM Amb544925 for 2 h, and then cell viability and caspase-3/7 activity were measured simultaneously. Cells were preincubated with 30 μM Amb544925 for 2 h, and then SMPD4 mRNA expression was measured by real-time PCR. The expression level of SMPD4 mRNA was expressed as the ratio of the expression level of Amb544925 treatment to that of the vehicle control. (**C**) Effect of Amb544925 on SMPD4 protein levels in HSC-3 cells (*n* = 4). Cells were preincubated with 30 μM Amb544925 for 2 h, and then whole cell proteins were extracted. Representative immunoblots and densitometric analysis for SMPD4 in lysates of HSC-3 cells treated with 30 μM Amb544925 for 2 h. Each column represents the densitometric measurement of SMPD4 protein normalized to GAPDH. The densitometric data are presented as the means ± SD (*n* = 4). ** *p* < 0.01 compared to the vehicle control.

Inhibition of CTL1-mediated choline uptake reduces the concentration of choline in the cell and inhibits the Kennedy pathway. Cancer cells actively divide and activate sphingomyelinase to break down sphingomyelin in the plasma membrane and cleave phosphocholine (PC) to maintain phospholipid synthesis by means of the Kennedy pathway [11,12]. Therefore, we investigated the effect of Amb544925 on the mRNA expression of various sphingomyelinase isoforms using RT-PCR. HSC-3 cells had the highest mRNA expression of SMPD4 among the sphingomyelinase isoforms (Figure 3A). When Amb544925 was exposed to HSC-3 cells for 2 h, the mRNA and protein levels of SMPD4 were increased (Figure 3B,C).

### 3.4. Effect of C2 Ceramide on Cell Viability and Caspase-3/7 Activity

**Figure 4:**
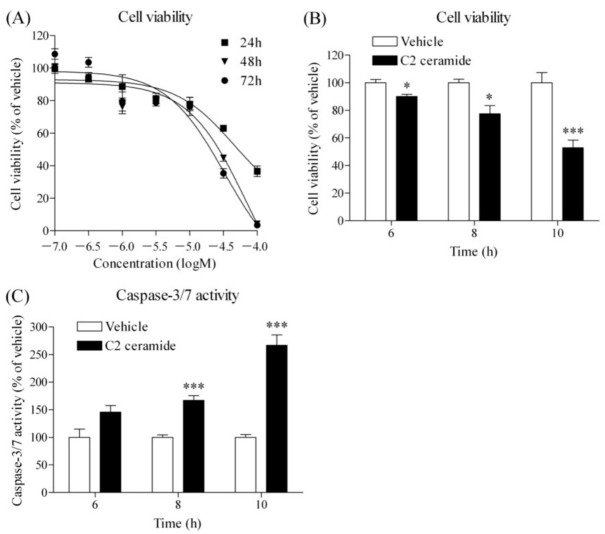
Effects of C2 ceramide on cell viability and caspase-3/7 activity in HSC-3 cells. (**A**) Effect of C2 ceramide on cell viability (*n* = 3). Cells were preincubated with each concentration of C2 ceramide for 24, 48, and 72 h, and then cell viability was measured. Results are presented as a percentage of the vehicle control. Effect of 50 µM C2 ceramide on cell viability (**B**) and caspase-3/7 activity (**C**) in HSC-3 cells (*n* = 4). Cells were incubated with 50 µM C2 ceramide for 6, 8, and 10 h, and then cell viability and caspase-3/7 activity were measured simultaneously. Results are presented as a percentage of the vehicle control. * *p* < 0.05 and *** *p* < 0.005 compared to the vehicle control.

As mentioned earlier, inhibition of choline uptake inhibits the Kennedy pathway, causing cancer cells to activate sphingomyelin metabolism and maintain phospholipid synthesis. At this time, PC and ceramide are released from sphingomyelin at the same time. Ceramide is known to cause the release of pro-apoptotic proteins from mitochondria and induce cell death by apoptosis [18,19]. Therefore, we investigated the effects of cell-membrane-permeable C2 ceramide on cell survival and caspase-3/7 activity in HSC-3 cells. C2 ceramide inhibited cell viability in a concentration- and time-dependent manner (Figure 4A). The effects of C2 ceramide on cell viability (Figure 4B) and caspase-3/7 activity (Figure 4C) at treatment times of 6, 8, and 10 h were examined simultaneously. C2 ceramide significantly inhibited the cell viability of HSC-3 cells and significantly increased caspase-3/7 activity, and both effects were inversely correlated.

### 3.5. Effect of Amb544925 and C2 Ceramide on the Expression of Survivin

**Figure 5:**
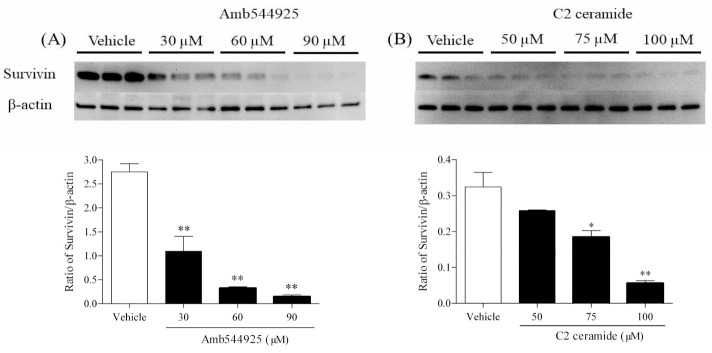
Effect of Amb544925 and C2 ceramide on survivin expression in HSC-3 cells. (**A**) Effect of Amb544925 on survivin expression in HSC-3 cells (*n* = 3). Cells were preincubated with 30, 60 and 90 μM Amb544925 for 8 h and then whole cell proteins were extracted. Representative immunoblots and densitometric analysis for survivin in lysates of HSC-3 cells treated with 30, 60 and 90 μM Amb544925 for 8 h. Each column represents the densitometric measurement of survivin protein normalized to β-actin. The densitometric data are presented as the means ± SD (*n* = 3). ** *p* < 0.01 compared to the vehicle control. (**B**) Effect of C2 ceramide on survivin expression in HSC-3 cells (*n* = 3). Cells were preincubated with 50, 75 and 100 μM C2 ceramide for 8 h and then whole cell proteins were extracted. Representative immunoblots and densitometric analysis for survivin in lysates of HSC-3 cells treated with 50, 75 and 100 μM C2 ceramide for 8 h. Each column represents the densitometric measurement of survivin protein normalized to β-actin. The densitometric data are presented as the means ± SD (*n* = 3). * *p* < 0.05 and ** *p* < 0.01 compared to the vehicle control.

Survivin is a member of the apoptosis suppressor family and is involved in the suppression of apoptosis and regulation of the cell cycle [20], and is highly expressed in OSCC. In contrast, the normal oral epithelium did not express survivin [21]. We therefore examined the effect of Amb544925 on survivin expression and whether the alteration of survivin expression is involved in the induction of apoptosis by C2 ceramide. The treatment of HSC-3 cells with 30, 60 or 90 µM Amb544925 for 8 h dramatically suppressed survivin expression in a concentration-dependent manner (Figure 5A). Similarly, 50, 75 or 100 µM C2 ceramide dramatically suppressed survivin expression after 8 h of treatment (Figure 5B).

### 3.6. Effect of Amb544925 and C2 Ceramide on the Migration of HSC-3 Cells

**Figure 6:**
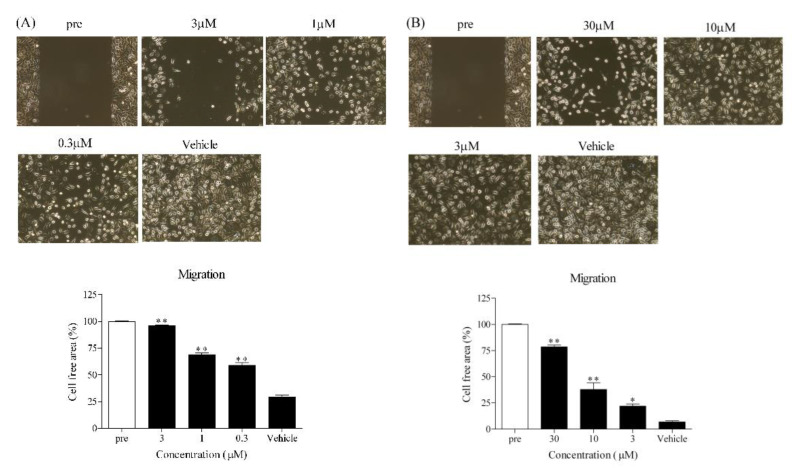
Effect of Amb544925 and C2 ceramide on HSC-3 cell migration. Cell migration assays were performed using culture-inserts. After cells were incubated with various concentrations of Amb544925 (**A**) and C2 ceramide (**B**) for 24 h, images of cell-free areas were acquired by digital microscopy (*n* = 4). Cell-free area (%) is expressed as percentage reduction relative to the cell-free area before drug administration (pre). * *p* < 0.05 and ** *p* < 0.01 compared to the vehicle control.

TSCC is known to have a high incidence of lymph node metastasis. Therefore, we investigated the effect of Amb544925 on migration ability, which is one of the indicators of metastasis. Amb544925 (0.3, 1, and 3 µM) significantly inhibited the migration of HSC-3 cells in a concentration-dependent manner (Figure 6A). C2 ceramide (3, 10 and 30 µM) also significantly inhibited the migration of HSC-3 cells as well as Amb544925 (Figure 6B).

### 3.7. Effect of Amb544925 in HSC-3 Cell Xenograft Model Mice

**Figure 7:**
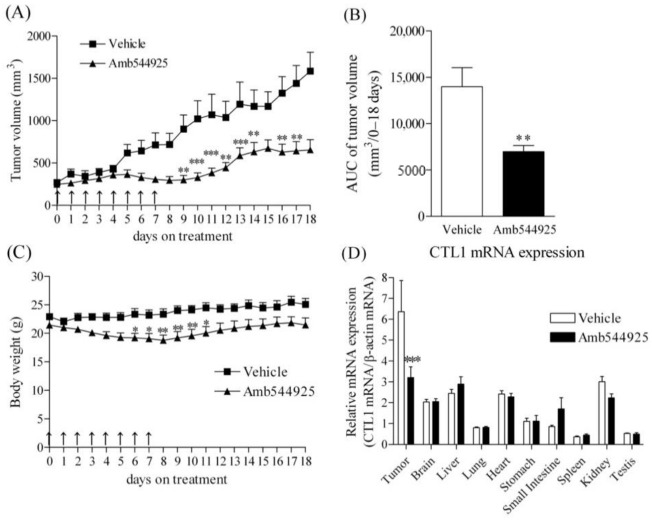
Effect of Amb544925 on tumor growth, body weight changes, and CTL1 mRNA expression in the HSC-3 human tongue cancer xenograft mouse model. (**A**) Tumor volumes were measured after intraperitoneal administration of 10 mg/kg Amb544925 (*n* = 8 mice) and vehicle control (*n* = 8 mice). The date of drug administration is indicated by an arrow. ** *p* < 0.01 and *** *p* < 0.005 compared to the vehicle control. (**B**) Area under curve (AUC) of tumor volume in each group over 0–18 days (*n* = 8 mice). ** *p* < 0.01 compared to the vehicle control. (**C**) The body weight of mice was measured at the time of drug administration (*n* = 8 mice). * *p* < 0.05 and ** *p* < 0.01 compared to the vehicle control. (**D**) Effect of Amb544925 on CTL1 mRNA expression in the tumor, brain, liver, lung, heart, stomach, small intestine, spleen, kidney, and testicles in the HSC-3 human tongue cancer xenograft mouse model. Relative mRNA expression is expressed as the ratio of CTL1 mRNA to β-actin mRNA in each tissue (*n* = 8 mice). *** *p* < 0.005 compared to the vehicle control.

The antitumor effect of Amb544925 in vivo was investigated in HSC-3 cell xenograft model mice. Intraperitoneal administration of 10 mg/kg Amb544925 for 8 consecutive days significantly suppressed the increase in tumor volume (Figure 7A). The AUC of tumor volume for 0–8 days was also significantly suppressed (Figure 7B). Continuous administration of Amb544925 resulted in a slight decrease (maximum 11%) in body weight compared to the control group, which improved after the administration (Figure 7C). Quantification of CTL1 mRNA expression in tumor tissues and organs showed that CTL1 mRNA expression in tumor tissues was significantly suppressed by Amb544925 administration. On the other hand, the expression of CTL1 mRNA in each organ was not affected by Amb544925 administration (Figure 7D).

### 3.8. Effect of Amb544925 and FBS on CTL1 mRNA Expression in HSC-3 Cells

**Figure 8:**
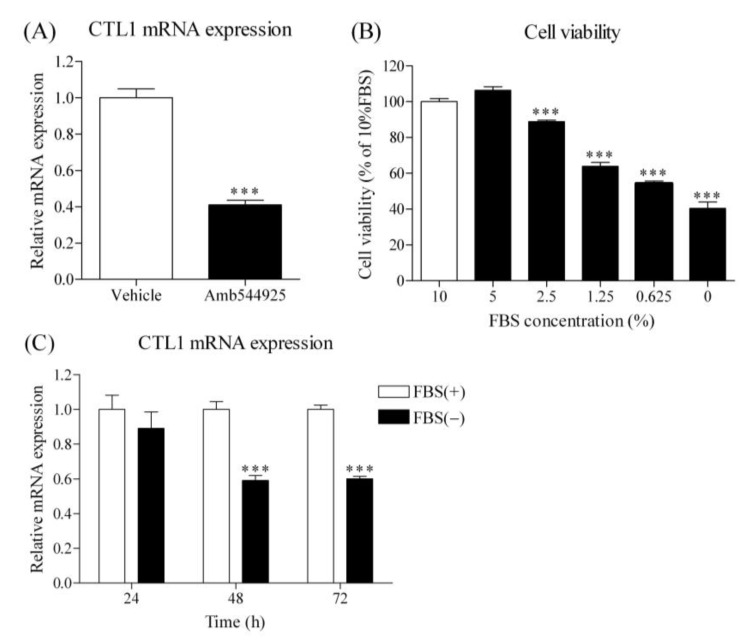
Effect of Amb544925 and FBS on CTL1 mRNA expression in HSC-3 cells. (**A**) Cells were incubated with 10 μM Amb544925 for 24 h, and then CTL1 mRNA expression was measured by real-time PCR (*n* = 4). The expression level of CTL1 mRNA was expressed as the ratio of the expression level of Amb544925 treatment to that of the vehicle control. *** *p* < 0.005 compared to the vehicle control. Effect of FBS on cell viability (**B**) and CTL1 mRNA expression (**C**) in HSC-3 cells (*n* = 4). The cell viability of HSC-3 cells cultured in medium containing various concentrations of FBS for 48 h was measured. Results are given as a percentage of cell viability measured in the presence of 10% FBS. *** *p* <0.005 compared to 10% FBS. (**C**) Effect of FBS on CTL1 mRNA expression in HSC-3 cells (*n* = 4). Cells were cultured in the presence (FBS+) and absence (FBS−) of 10% FBS for 24, 48, and 72 h, and then CTL1 mRNA expression was analyzed by real-time PCR. *** *p* < 0.005 compared to 10% FBS (FBS+).

In a mouse model of HSC-3 cell xenograft, administration of Amb544925 suppressed CTL1 mRNA expression, suggesting that inhibition of cell proliferation may be linked to CTL1 expression. Therefore, we examined the effect of Amb544925 on CTL1 mRNA expression in the culture system of HSC-3 cells. Treatment with 10 µM Amb544925 for 24 h significantly suppressed CTL1 mRNA expression (Figure 8A). Next, the effect of FBS was examined in order to clarify the relationship between cell proliferation and CTL1 expression. Cell survival was inhibited by decreasing the concentration of FBS in the culture medium (Figure 8B). The expression of CTL1 mRNA was significantly decreased at 48 and 72 h when the cells were cultured in the medium without FBS (Figure 8C).

### 3.9. Effect of HC-3 on Cell Viability, Caspase-3/7 Activity and CTL1 mRNA Expression in HSC-3 Cells

**Figure 9:**
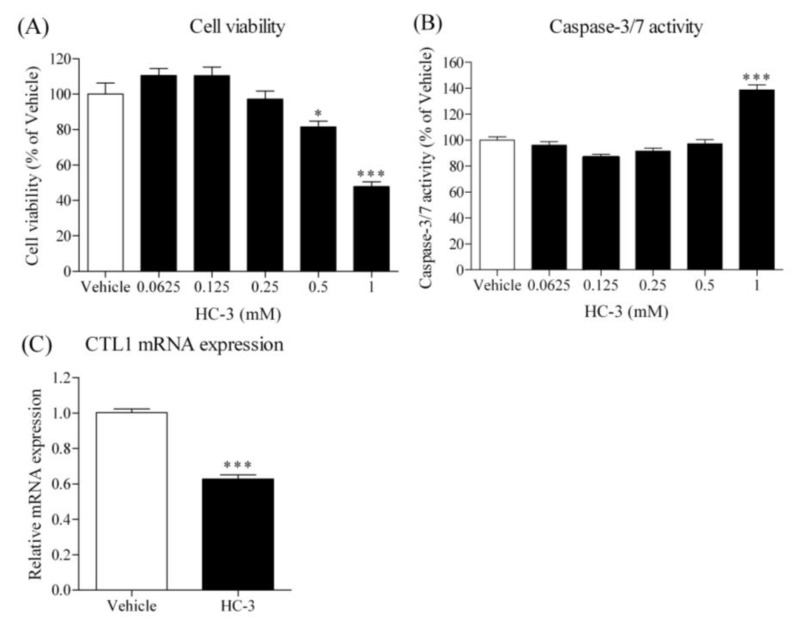
Effect of choline uptake inhibitor, HC-3, on cell viability, caspase-3/7 activity and CTL1 mRNA expression in HSC-3 cells. Cells were incubated with each concentration of HC-3 for 72 h, and then cell viability (**A**) and caspase-3/7 activity (**B**) were measured (*n* = 4). * *p* < 0.05 and *** *p* < 0.005 compared to the vehicle control (Normal). (**C**) Cells were incubated with 1 mM HC-3 for 72 h, and then CTL1 mRNA expression was analyzed by real-time PCR (*n* = 4). *** *p* < 0.005 compared to the vehicle control (Normal).

Hemicolinium-3 (HC-3) is a CTL1-mediated choline uptake inhibitor, and its effect in cancer cells was previously reported [13,15,16]. The effect of HC-3 on the viability of HSC-3 cells was examined, and it inhibited the cell viability in a concentration-dependent manner (Figure 9A). On the other hand, a high concentration of HC-3 (1 mM) significantly increased caspase-3/7 activity (Figure 9B). Furthermore, HC-3 significantly decreased the expression of CTL1 mRNA in HSC-3 cells (Figure 9C).

## 4. Discussion

Surgery is the initial treatment of choice for most TSCC, but radiation therapy or chemoradiation may be added after surgery if the disease is relatively advanced or has high-risk features. In addition, since lymph node metastasis halves the survival rate, adjuvant therapy in combination with radiation and chemotherapy is recommended for patients with advanced lymph node metastasis [22]. The most common anticancer drug used is 5-fluorouracil plus cisplatin, which is a classic anticancer drug with strong side effects. It is desirable to develop new therapeutic mechanisms and drugs that inhibit lymph node metastasis instead of conventional anticancer drugs.

Cancer cells have an abnormally high uptake of choline, which is used for cell membrane synthesis to maintain active cell division [10]. Using these characteristics of cancer cells, positron emission tomography (PET) imaging using ^14^C-choline and ^18^F-choline has been performed in various cancer patients [7,8,9]. We previously reported that choline uptake in HSC-3 cells involves a single transporter and that CTL1 is expressed on the plasma membrane [13]. In addition, choline uptake in HSC-3 cells resembles the transport properties of CTL1. Therefore, we conclude that CTL1 is functionally expressed in HSC-3 cells, and inhibition of its function causes apoptotic cell death [13]. We have also shown that choline uptake in many cancer cell lines is mediated by CTL1 [15,16,23,24]. Thus, the CTL1-mediated choline uptake mechanism may be a target for novel cancer therapies.

Currently, there are several groups of plant-derived anticancer drugs. The first group includes vincristine and vinblastine, which are vinca alkaloids that inhibit the polymerization of microtubules that are important in cell division and stop cell division. The second group includes the taxanes paclitaxel and docetaxel, which inhibit microtubule depolymerization. A third group includes irinotecan and etoposide, which inhibit the action of topoisomerase. These plant-derived anticancer drugs are used to treat many types of cancer and have shown high efficacy. Therefore, we screened compounds that inhibit CTL1 and have antitumor activity in HSC-3 cells using a plant-derived organic compound library. As a result of our screening, we discovered an isoquinoline derivative, Amb544925, which inhibits choline uptake and inhibits cell proliferation. Amb544925 is a non-cationic compound, unlike the previously reported cationic compounds Amb4269951 and Amb4269675, which have a dimethyl group [11,12]. It seems to be more stable and easier to synthesize than both Amb4269951 and Amb4269675. Lipinski et al. (1997) compared the list of drug candidates with the list of conventional organic compounds and summarized the chemical properties of compounds that are likely to be orally available as a “rule of five” (i.e., a molecule with a molecular weight less than 500, no more than 5 hydrogen bond donors (OH, NH), no more than 10 hydrogen bond acceptors (N, O, etc), and a logP not greater than 5) [25]. Compounds that do not fit into either of these two categories are poorly absorbed, making it difficult for them to eventually become pharmaceuticals. Am544925 satisfies all of these chemical properties and is suitable as a lead compound for pharmaceuticals.

Firstly, we examined the relationship between the inhibitory effect of Am544925 on CTL1-mediated choline uptake and cell survival and caspase activity in HSC-3 cells. The concentration ranges of choline uptake inhibition and cell viability inhibition were completely the same. In addition, cell survival was inhibited in a time-dependent manner, while caspase-3/7 activity was increased, showing an inverse correlation. Previous studies have shown that choline uptake inhibitors and choline deficiency inhibited cell viability and increased caspase-3/7 activity in various cancer cell lines [11,12,13,15,16]. These results suggest that the inhibition of choline uptake by Am544925 increased caspase-3/7 activity and induced apoptotic cell death.

Based on our previous studies, we proposed the following mechanism of apoptosis caused by inhibition of choline uptake via CTL1 [11,12]. Inhibition of CTL1-mediated choline uptake leads to a decrease in intracellular choline resulting in decreased PC synthesis, which prevents the production of PCho, a component of the cell membrane. As a result, cancer cells are unable to proliferate, so they try to maintain cell growth by activating sphingomyelinase to break down sphingomyelin, which is also a component of the cell membrane, and produce PC. At the same time that PC is produced, ceramide, an apoptosis-inducing molecule, is produced from sphingomyelin, and cell death by apoptosis is induced. Therefore, we investigated whether the antitumor effect of Am544925 in HSC-3 cells is mediated by these mechanisms. There are several isoforms of sphingomyelinase, the enzyme that produces PC and ceramide from sphingomyelin [26]. Many cancers alter the metabolism of sphingolipids, leading to a decrease in ceramide, a pro-apoptotic lipid, and an increase in sphingosine-1 phosphate, a proliferative lipid. These changes are thought to affect the carcinogenicity and metastatic potential of cancer [27]. We examined the expression of sphingomyelinase isoforms and found that sphingomyelin phosphodiesterase 4 (SMPD4) was mainly highly expressed in HSC-3 cells, which is considered to be the main enzyme involved in ceramide production. Am544925 significantly increased the mRNA and protein expression of SMPD4. These results suggest that Amb544925 increases the expression of SMPD4 to produce ceramide, an apoptosis-inducing molecule. Unfortunately, we do not have analytical techniques such as LC-MS/MS to quantify intracellular ceramides, so we used cell membrane-permeable C2 ceramide to investigate its effect on cell survival and caspase activity. C2 ceramide showed a similar effect to Amb544925 in inhibiting cell viability and increasing caspase-3/7 activity in HSC-3 cells. These results suggest that the antitumor effect of Amb544925 is mediated by ceramide. Ceramide has been shown to induce apoptosis and autophagy in cancer cells and has become a focus of cancer therapy in terms of proliferation, carcinogenesis, and metastasis in liver and colon cancer [28,29,30]. In head and neck squamous cell carcinoma cells, C2 ceramide exerts its antitumor effects by inducing apoptosis and necrosis, and these cytotoxic effects are enhanced by autophagy inhibitors [31]. In addition, chemotherapy drugs such as doxorubicin and gemcitabine have been reported to increase intracellular ceramide levels in head and neck cancers [32].

Next, we focused on survivin, a member of the inhibitor of apoptosis (IAP) family of proteins. Survivin is known to be expressed at high levels in most common cancers and almost no expression in fully differentiated normal cells and inhibits apoptosis of cancer cells by suppressing caspases [33,34]. High expression of survivin in cancer cells is associated with tumor cell growth and progression, resistance to therapy, and poor prognosis, suggesting a therapeutic strategy targeting survivin [35]. We investigated whether survivin was involved in the antitumor effect of Amb544925 and C2 ceramide, and both of them drastically suppressed the expression of survivin in HSC-3 cells. These results indicate that when CTL1-mediated choline uptake is inhibited by Amb544925, SMPD4 expression is increased, and ceramide is produced in the cell. The increased ceramide may induce apoptosis by suppressing survivin expression. Anticancer drugs with such a novel mechanism may be effective against cancers that have acquired resistance to treatment with anticancer drugs. In the future, it will be necessary to verify the effects of the combination with existing drugs.

TSCC is associated with invasive lesions and perineural proliferation. Additionally, recurrence rates are high and lymph node metastatic tumors are frequent in approximately 40% of oral cancer patients [36]. Therefore, multidisciplinary treatment interventions for cervical lymph node metastases are essential for the treatment of TSCC. We investigated whether Amb544925 has not only antitumor effects but also anti-metastatic effects. Cell migration and invasion play an important role in tumor metastasis, and migration ability is a major component of metastasis. Amb544925 significantly inhibited the migration of HSC-3 cells at concentrations (0.3 and 1 µM) that slightly inhibited cell survival. Since Amb544925 inhibits the migration of HSC-3 cells at low concentrations, it is expected to inhibit metastasis to lymph nodes in the pharmacotherapy of TSCC patients. Several studies have shown that knockdown of survivin with siRNA or shRNA reduces cancer cell migration and invasion. In addition, YM155, a small-molecule survivin inhibitor, suppressed lymph node metastasis in a xenograft animal model [37,38]. Ceramide also significantly inhibited the migration of HSC-3 cells as well as Amb544925. These results suggest that Amb544925 inhibits TSCC proliferation and lymph node metastasis via the ceramide/survivin pathway.

In xenograft animal models, Amb544925 significantly inhibited tumor growth and slightly affected weight gain, but other behaviors and responses to handling were normal. At autopsy, there were no major abnormalities in any organs. Mice treated with cisplatin, which is used clinically to treat cancer, showed severe weight loss and hypothermia, as well as the induction of mechanical and cold allodynia [39]. Therefore, Amb544925 does not appear to cause serious side effects, in contrast to existing drugs such as cisplatin.

Interestingly, the mRNA expression of CTL1 in various organs of Amb544925-treated mice did not differ from that of control mice, whereas it was significantly decreased in tumor tissues. These results suggest that suppression of CTL1 expression may be involved in the antitumor activity of Amb544925 in xenograft animal models. Therefore, the relationship between the suppression of CTL1 expression by Amb544925 and the suppression of cell proliferation was verified in an in vitro culture system. In HSC-3 cells, Amb544925 also significantly suppressed CTL1 mRNA expression, suggesting that the function of CTL1 may be closely related to cell proliferation. In cell culture systems, cell proliferation is stimulated by FBS-derived growth factors. In HSC-3 cell culture, cell survival was inhibited by decreasing FBS concentration, and CTL1 expression was significantly decreased when FBS was removed. Furthermore, the inhibition of choline uptake by HC-3, a choline uptake inhibitor, decreased cell viability and CTL1 expression, suggesting that the decrease in cell viability caused by choline uptake inhibition is involved in the suppression of CTL1 expression. These results suggest that Amb544925 inhibited cell proliferation through the ceramide/survivin pathway by inhibiting choline uptake, resulting in decreased CTL1 expression. This decrease in CTL1 expression is also thought to be involved in the suppression of cell proliferation.

## 5. Conclusions

Amb544925, a plant-derived organic compound, may lead to the development of novel anticancer agents targeting CTL1, which exerts its antitumor effects through the ceramide/survivin pathway, a novel mechanism of action. It also has an inhibitory effect on the migration of cancer cells and is expected to suppress cervical lymph node metastasis in TSCC patients.

## Data Availability

Materials described in the manuscript, including all relevant raw data, will be freely available to any scientist wishing to use them for non-commercial purposes upon request via e-mail to the corresponding author.

## Supplementary Materials

The following supporting information can be downloaded athttps://www.mdpi.com/article/10.3390/cancers14020329/s1, Figure S1: Effect of Amb544925 on cell viability in various cancer cell lines.
