# Supplementary material for: A Novel Plant-Derived Choline Transporter-like Protein 1 Inhibitor, Amb544925, Induces Apoptotic Cell Death via the Ceramide/Survivin Pathway in Tongue Squamous Cell Carcinoma
Source: Cancers (Basel). 2022 Jan 10;14(2):329. doi: 10.3390/cancers14020329 (PMC8773621; doi:10.3390/cancers14020329)
Supplement: Supplementary file 1 [file cancers-14-00329-s001.zip › supplementary/cancers-1481792-original image.pdf]

Fig.3C . Detection of SMPD4 and GAPDH proteins by Western blot analysis.

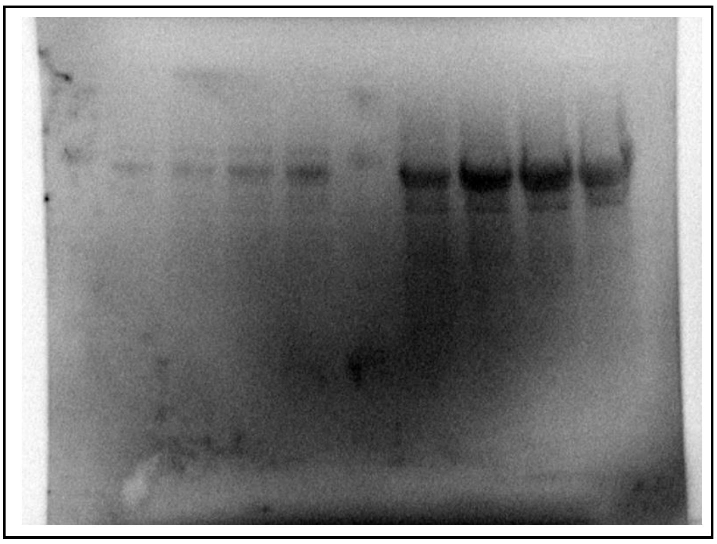

Lane 1: 5μL Prestained Molecular Weight Marker
Lane 2: 20μL lysates of HSC-3 cells with vehicle for 2 h
Lane 3: 20μL lysates of HSC-3 cells with vehicle for 2 h
Lane 4: 20μL lysates of HSC-3 cells with vehicle for 2 h
Lane 5: 20μL lysates of HSC-3 cells with vehicle for 2 h
Lane 6: 5μL Prestained Molecular Weight Marker
Lane 7: 20μL lysates of HSC-3 cells treated with 30μM Amb544925 for 2 h
Lane 8: 20μL lysates of HSC-3 cells treated with 30μM Amb544925 for 2 h
Lane 9: 20μL lysates of HSC-3 cells treated with 30μM Amb544925 for 2 h
Lane 10: 20μL lysates of HSC-3 cells treated with 30μM Amb544925 for 2 h

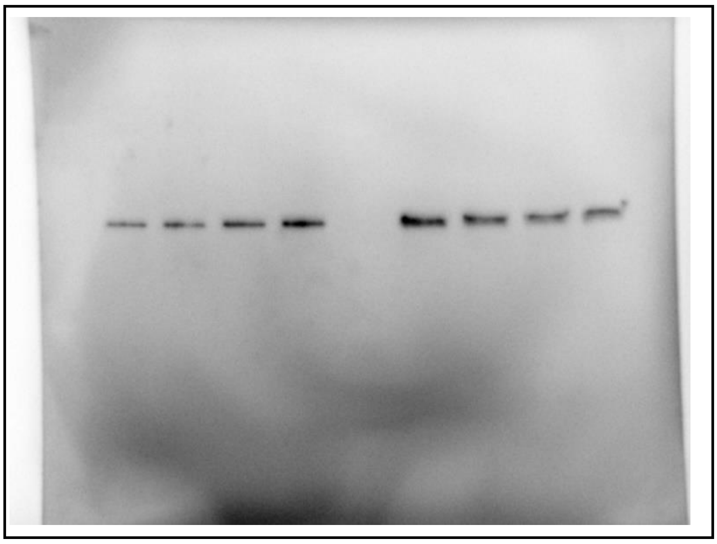

Lane 1: 5μL Prestained Molecular Weight Marker
Lane 2: 20μL lysates of HSC-3 cells with vehicle for 2 h
Lane 3: 20μL lysates of HSC-3 cells with vehicle for 2 h
Lane 4: 20μL lysates of HSC-3 cells with vehicle for 2 h
Lane 5: 20μL lysates of HSC-3 cells with vehicle for 2 h
Lane 6: 5μL Prestained Molecular Weight Marker
Lane 7: 20μL lysates of HSC-3 cells treated with 30μM Amb544925 for 2 h
Lane 8: 20μL lysates of HSC-3 cells treated with 30μM Amb544925 for 2 h
Lane 9: 20μL lysates of HSC-3 cells treated with 30μM Amb544925 for 2 h
Lane 10: 20μL lysates of HSC-3 cells treated with 30μM Amb544925 for 2 h

Fig.5A . Detection of Survivin and β-actin proteins
by Western blot analysis.

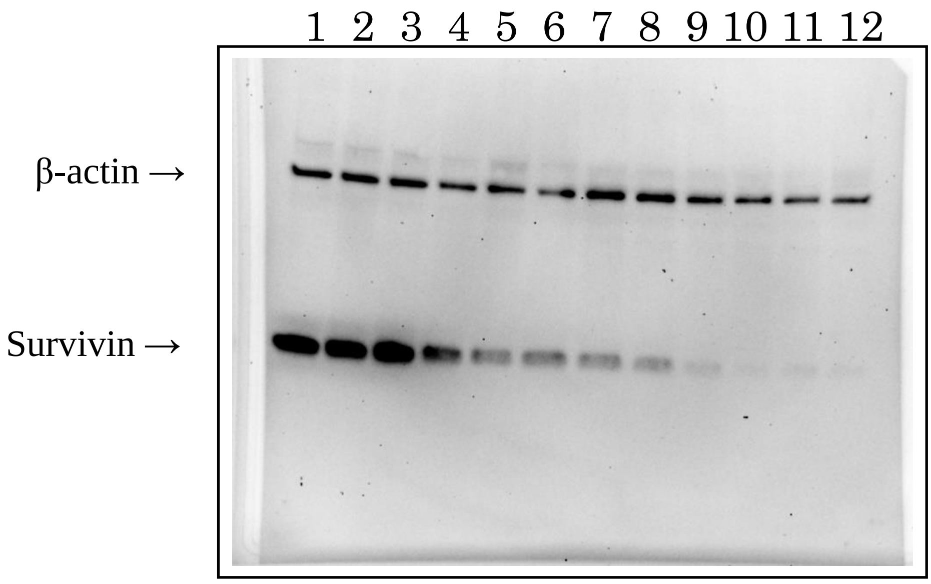

Lane 1: 20μL lysates of HSC-3 cells with Vehicle for 8 h
Lane 2: 20μL lysates of HSC-3 cells with Vehicle for 8 h
Lane 3: 20μL lysates of HSC-3 cells with Vehicle for 8 h
Lane 4: 20μL lysates of HSC-3 cells treated with 30μM Amb544925 for 8 h
Lane 5: 20μL lysates of HSC-3 cells treated with 30μM Amb544925 for 8 h
Lane 6: 20μL lysates of HSC-3 cells treated with 30μM Amb544925 for 8 h
Lane 7: 20μL lysates of HSC-3 cells treated with 60μM Amb544925 for 8 h
Lane 8: 20μL lysates of HSC-3 cells treated with 60μM Amb544925 for 8 h
Lane 9: 20μL lysates of HSC-3 cells treated with 60μM Amb544925 for 8 h
Lane 10: 20μL lysates of HSC-3 cells treated with 90μM Amb544925 for 8 h
Lane 11: 20μL lysates of HSC-3 cells treated with 90μM Amb544925 for 8 h
Lane 12: 20μL lysates of HSC-3 cells treated with 90μM Amb544925 for 8 h

Fig.5B . Detection of Survivin and β-actin proteins
by Western blot analysis.

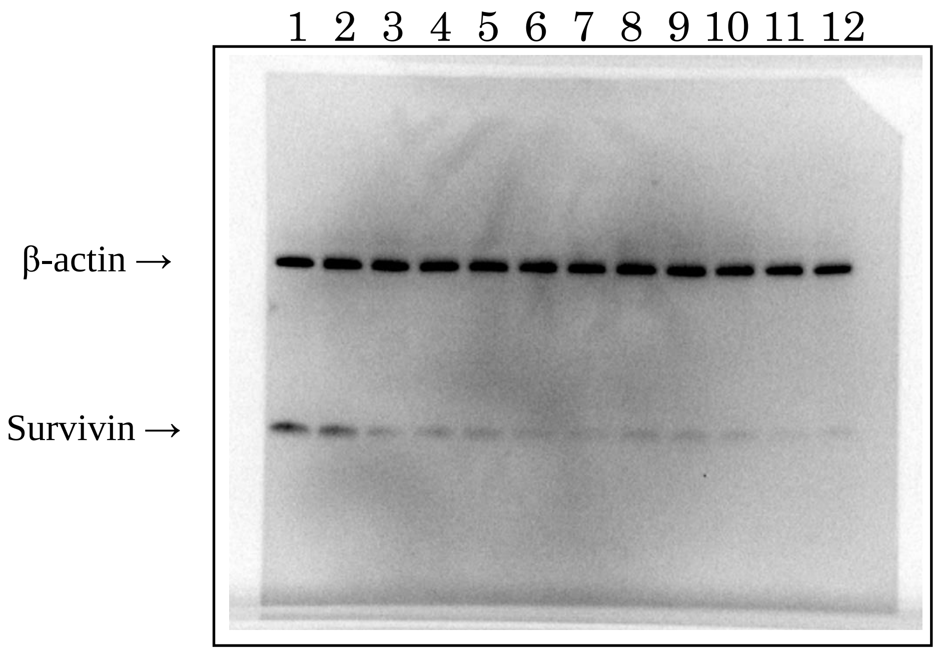

Lane 1: 20µL lysates of HSC-3 cells with Vehicle for 8 h
Lane 2: 20µL lysates of HSC-3 cells with Vehicle for 8 h
Lane 3: 20µL lysates of HSC-3 cells with Vehicle for 8 h
Lane 4: 20µL lysates of HSC-3 cells treated with 50µM C2 ceramide for 8 h
Lane 5: 20µL lysates of HSC-3 cells treated with 50µM C2 ceramide for 8 h
Lane 6: 20µL lysates of HSC-3 cells treated with 50µM C2 ceramide for 8 h
Lane 7: 20µL lysates of HSC-3 cells treated with 75µM C2 ceramide for 8 h
Lane 8: 20µL lysates of HSC-3 cells treated with 75µM C2 ceramide for 8 h
Lane 9: 20µL lysates of HSC-3 cells treated with 75µM C2 ceramide for 8 h
Lane 10: 20µL lysates of HSC-3 cells treated with 100µM C2 ceramide for 8 h
Lane 11: 20µL lysates of HSC-3 cells treated with 100µM C2 ceramide for 8 h
Lane 12: 20µL lysates of HSC-3 cells treated with 100µM C2 ceramide for 8 h
