# Supplementary material for: A Novel Plant-Derived Choline Transporter-like Protein 1 Inhibitor, Amb544925, Induces Apoptotic Cell Death via the Ceramide/Survivin Pathway in Tongue Squamous Cell Carcinoma
Source: Cancers (Basel). 2022 Jan 10;14(2):329. doi: 10.3390/cancers14020329 (PMC8773621; doi:10.3390/cancers14020329)
Supplement: Supplementary file 1 [file cancers-14-00329-s001.zip › supplementary/cancers-1481792-supplementary.pdf]

Supplementary Materials

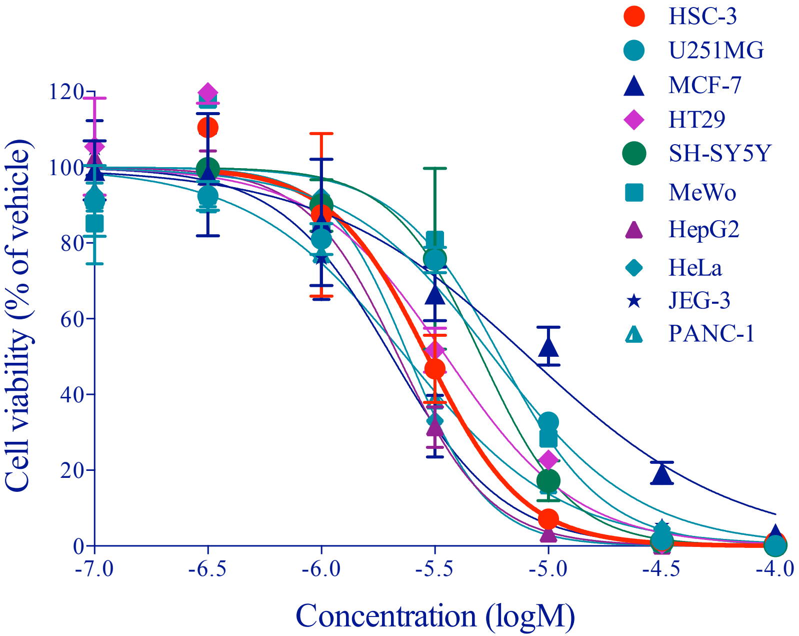

HSC-3	Oral squamous carcinoma
U251MG	Glioma
MCF-7	Breast cancer cells
HT29	Colon adenocarcinoma
SH-SY5Y	Neuroblastoma
MeWo	Melanoma
HepG2	Hepatocellular carcinoma
HeLa	Cervix adenocarcinoma
JEG-3	Choriocarcinoma
PANC-1	Pancreatic carcinoma cells

Figure S1: Effect of Amb544925 on cell viability in various cancer cell lines. Cells were pre-incubated with various concentrations of Amb544925 for 24h, and then the cells were counted. The results are given as a percentage of the findings in the vehicle control. Each point represents the mean ± S.D.(n=4).
